# Supplementary material for: Genome-Wide Identification and Multi-Stress Response Analysis of the DABB-Type Protein-Encoding Genes in Brassica napus
Source: Int J Mol Sci. 2024 May 24;25(11):5721. doi: 10.3390/ijms25115721 (PMC11171964; doi:10.3390/ijms25115721)
Supplement: Supplementary file 1 [file ijms-25-05721-s001.zip › Supplementary manuscript.pdf]

International Journal of
Molecular Sciences

MDPI

Article

Supplementary: Genome-wide identification and multi-stress response analysis of the DABB-type protein-encoding genes in *Brassica napus*

Siyi Wang †, Kunmei Wang †, Qi Xia and Shitou Xia *

Hunan Provincial Key Laboratory of Phytohormones and Growth Development, College of Bioscience and Biotechnology, Hunan Agricultural University, Changsha 410128, China; wangsiyi@stu.hunau.edu.cn (S.W.); yuhunan@stu.hunau.edu.cn (K.W.); xiaqi@stu.hunau.edu.cn (Q.X.)

* Correspondence: xstone0505@hunau.edu.cn (S.X.)

† These authors contributed equally to this work.

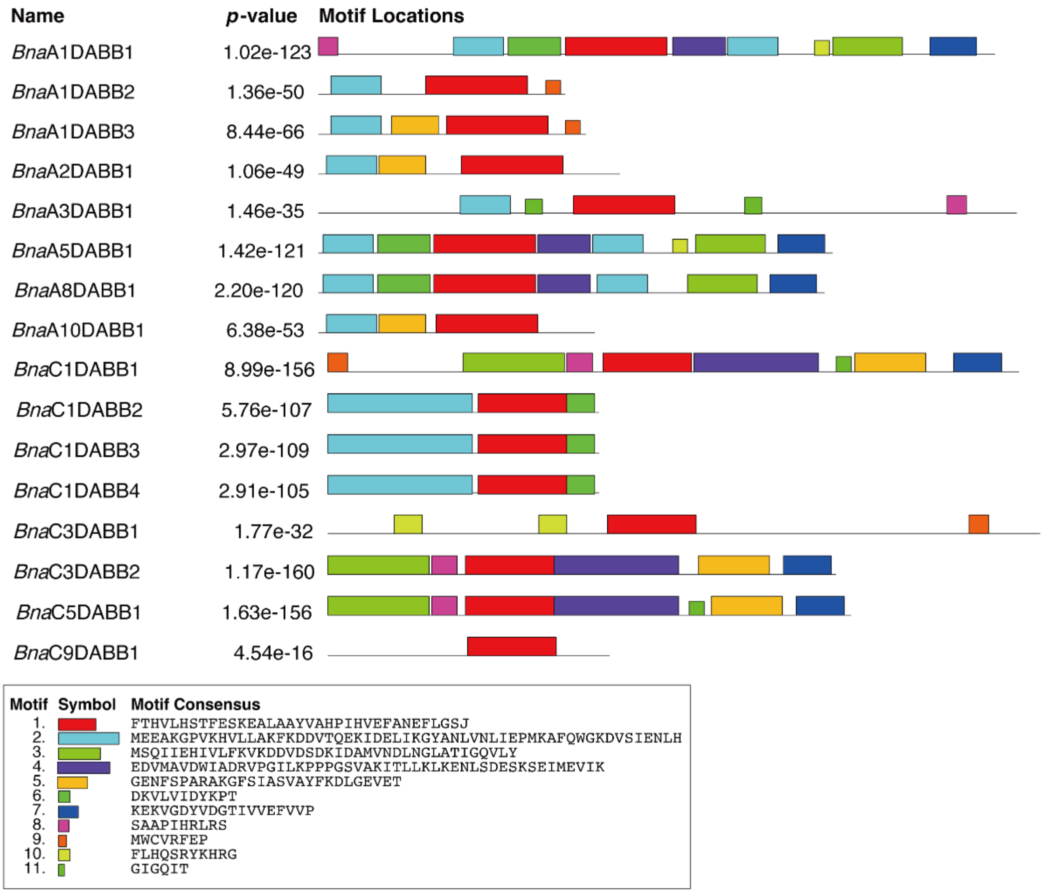

Figure S1. Motif locations analysis of *Bna*DABBs. Different color blocks represent different motifs.

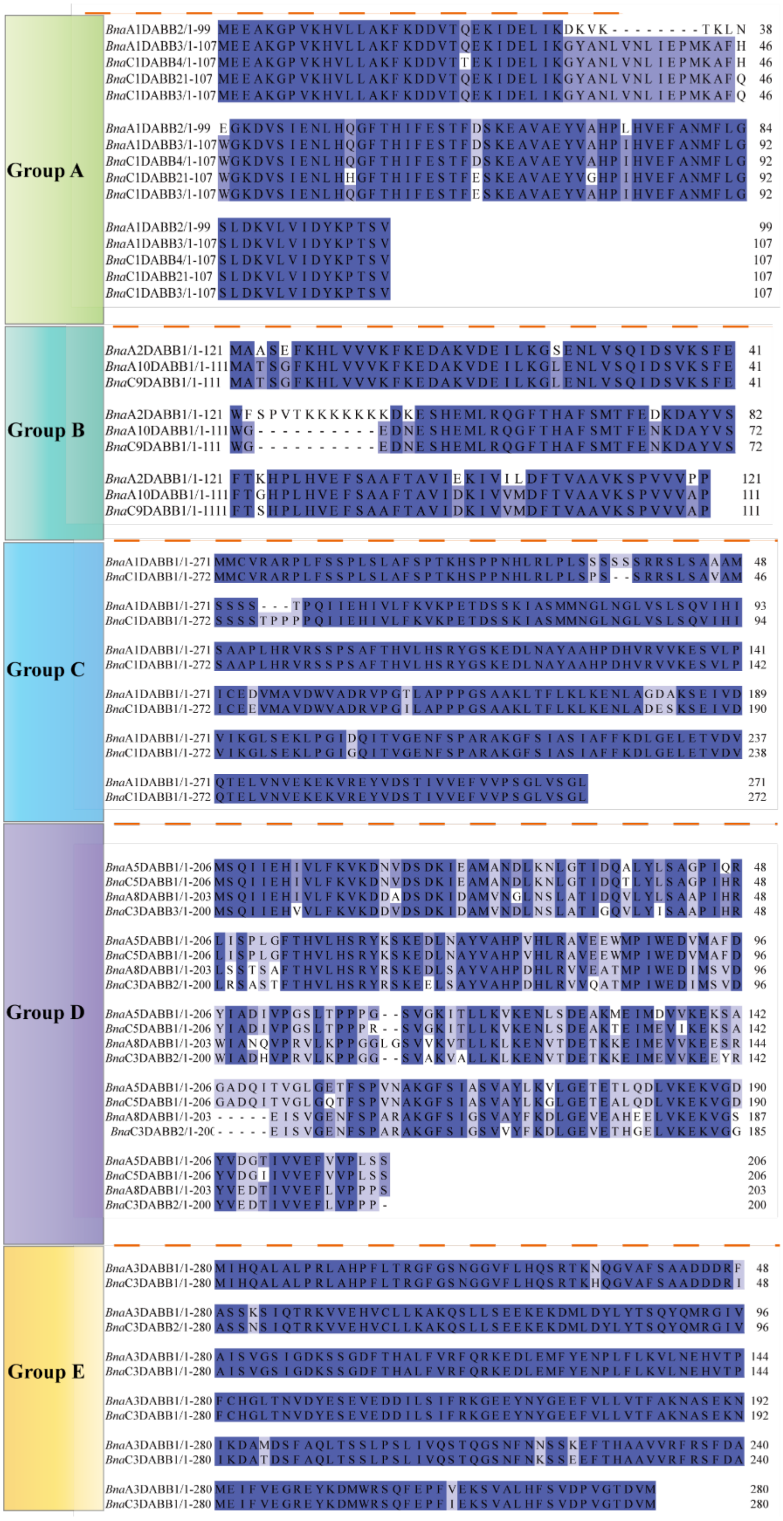

Figure S2. Protein sequence alignment analysis of *Bna*DABBs. Color represents the degree of conservatism of protein sites, and the darker the color, the more conservative it is.

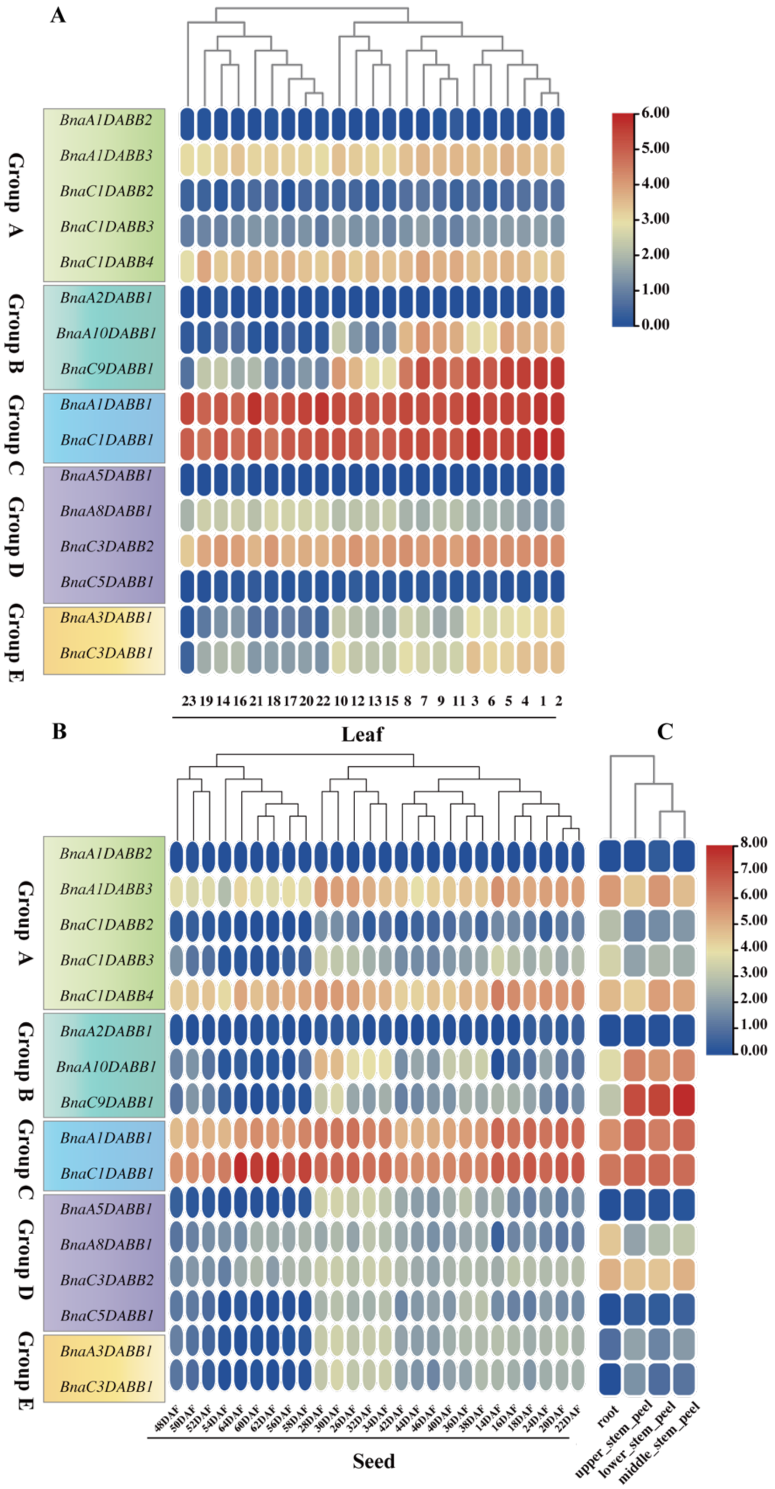

Figure S3. Analysis of expression patterns. (**A**)Analysis of expression patterns during leaf development stage. **(B)** Analysis of expression patterns during seed development stage. **(C)** Analysis of expression patterns root and stem. TPM values of *BnaDABBs* transformed by log2 were used in TBtools to construct heat maps. Expression levels depicted by the different colors on the scale. Red and blue represent high and low expression levels respectively.

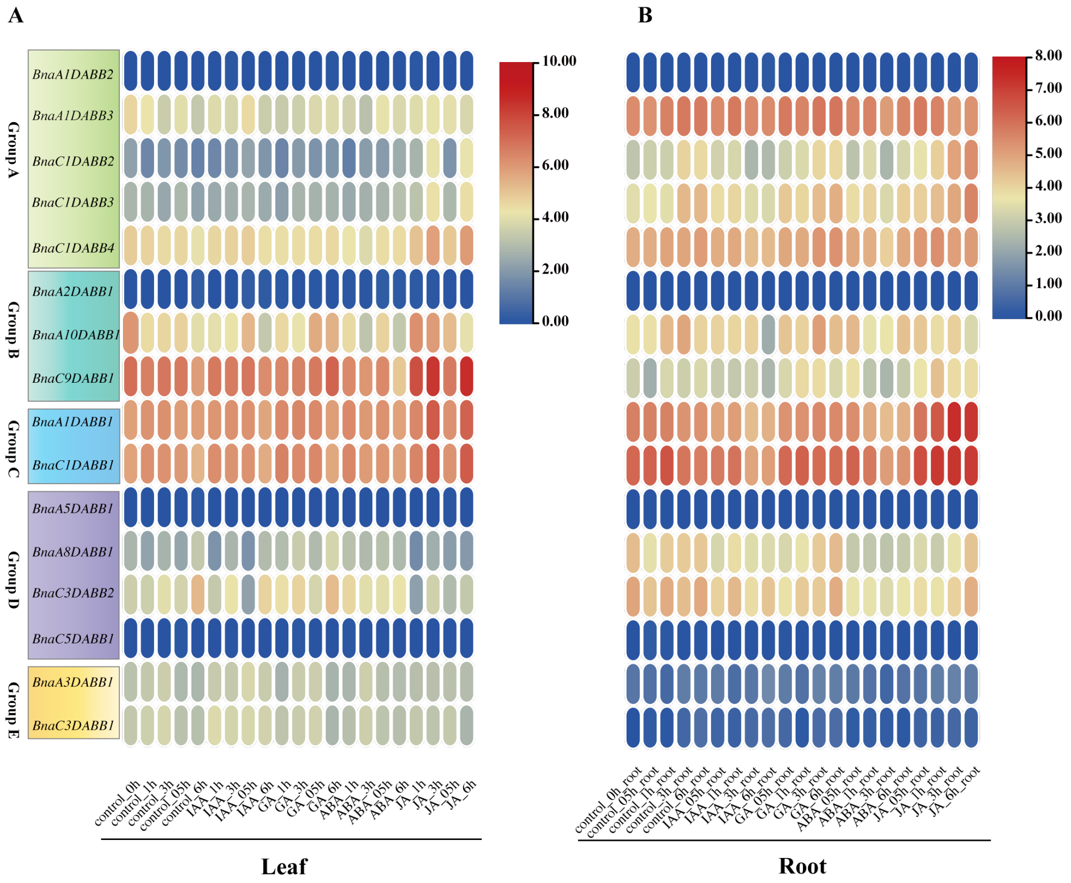

Figure S4. Expression profiling of *BnaDABB* genes under plant hormones treatment. **(A)** The expression profiling of *BnaDABBs* under IAA，GA，ABA，JA treatment in leaf. **(B)** The expression profiling of *BnaDABBs* under IAA，GA，ABA，JA treatment in root. TPM values of *BnaDABBs* transformed by log2 were used in TBtools to construct heat maps. Expression levels depicted by the different colors on the scale. Red and blue represent high and low expression levels respectively.

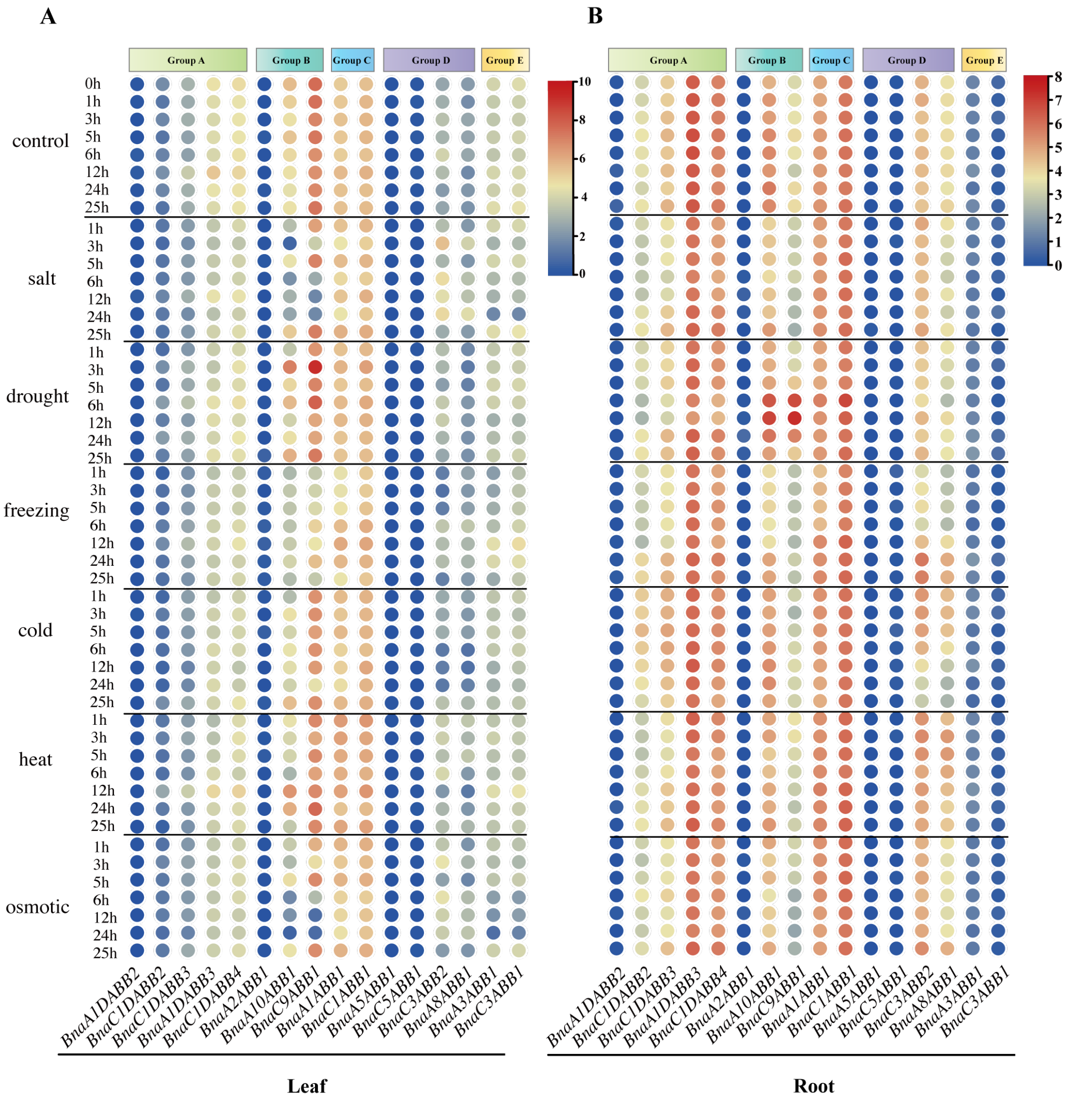

Figure S5. Expression profiling of *BnaDABBs* under abiotic stresses treatment. **(A)**Analysis of expression patterns of *BnaDABBs* in response to salt, drought, freezing, cold, heat, and osmotic treatments in leaves. **(B)** Analysis of expression patterns of *BnaDABBs* in response to salt, drought, freezing, cold, heat, and osmotic treatments in root. TPM values *BnaDABBs* transformed by log2 were used in TBtools to construct heat maps. Expression levels are depicted by the different colors on the scale. Red and blue represent high and low expression levels respectively.
