# Supplementary figures and images for: Genome-Wide Identification and Multi-Stress Response Analysis of the DABB-Type Protein-Encoding Genes in Brassica napus
Source: Int J Mol Sci. 2024 May 24;25(11):5721. doi: 10.3390/ijms25115721 (PMC11171964; doi:10.3390/ijms25115721)

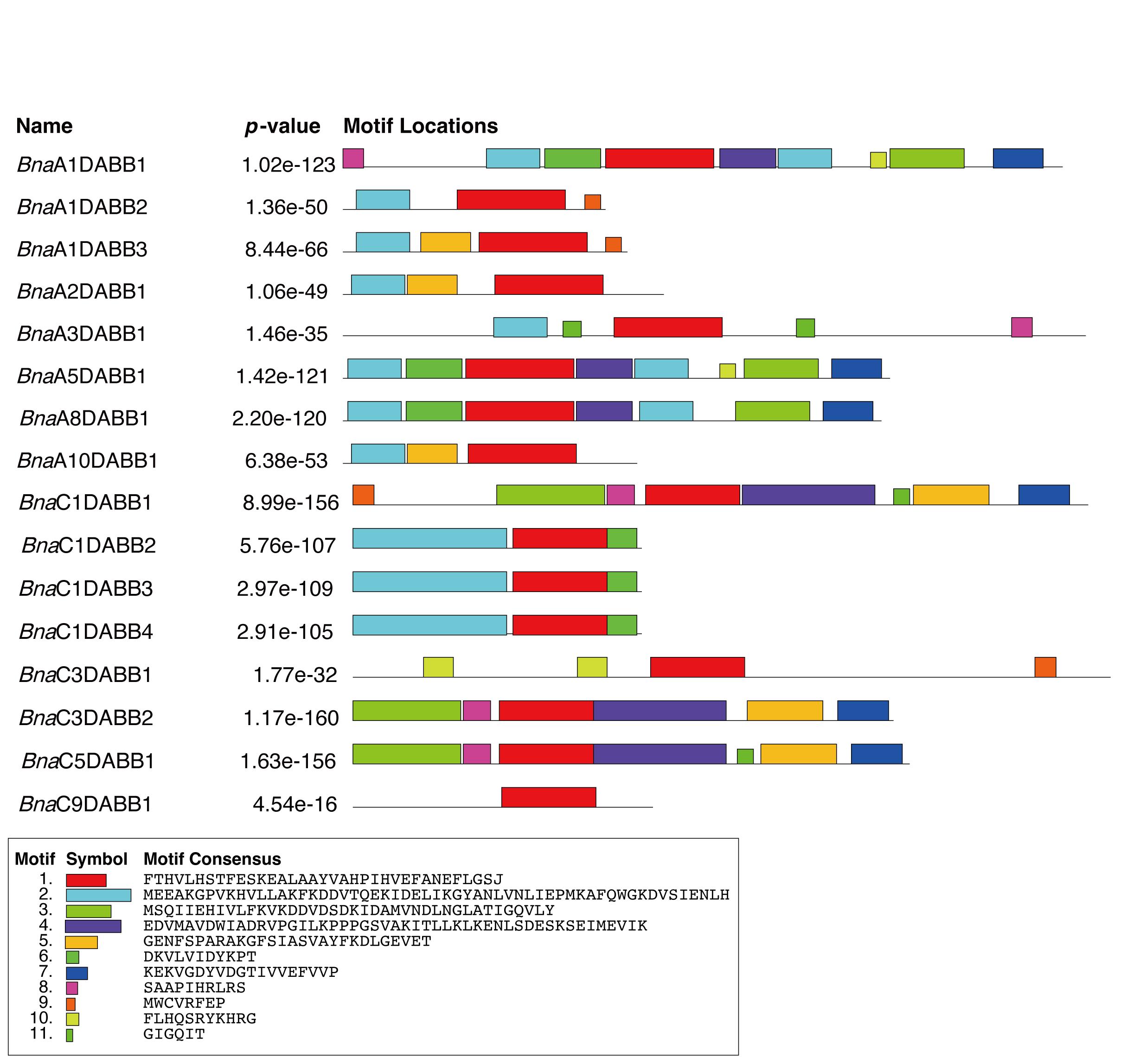

Supplement: Supplementary file 1 [file ijms-25-05721-s001.zip › Figure S1.jpg]

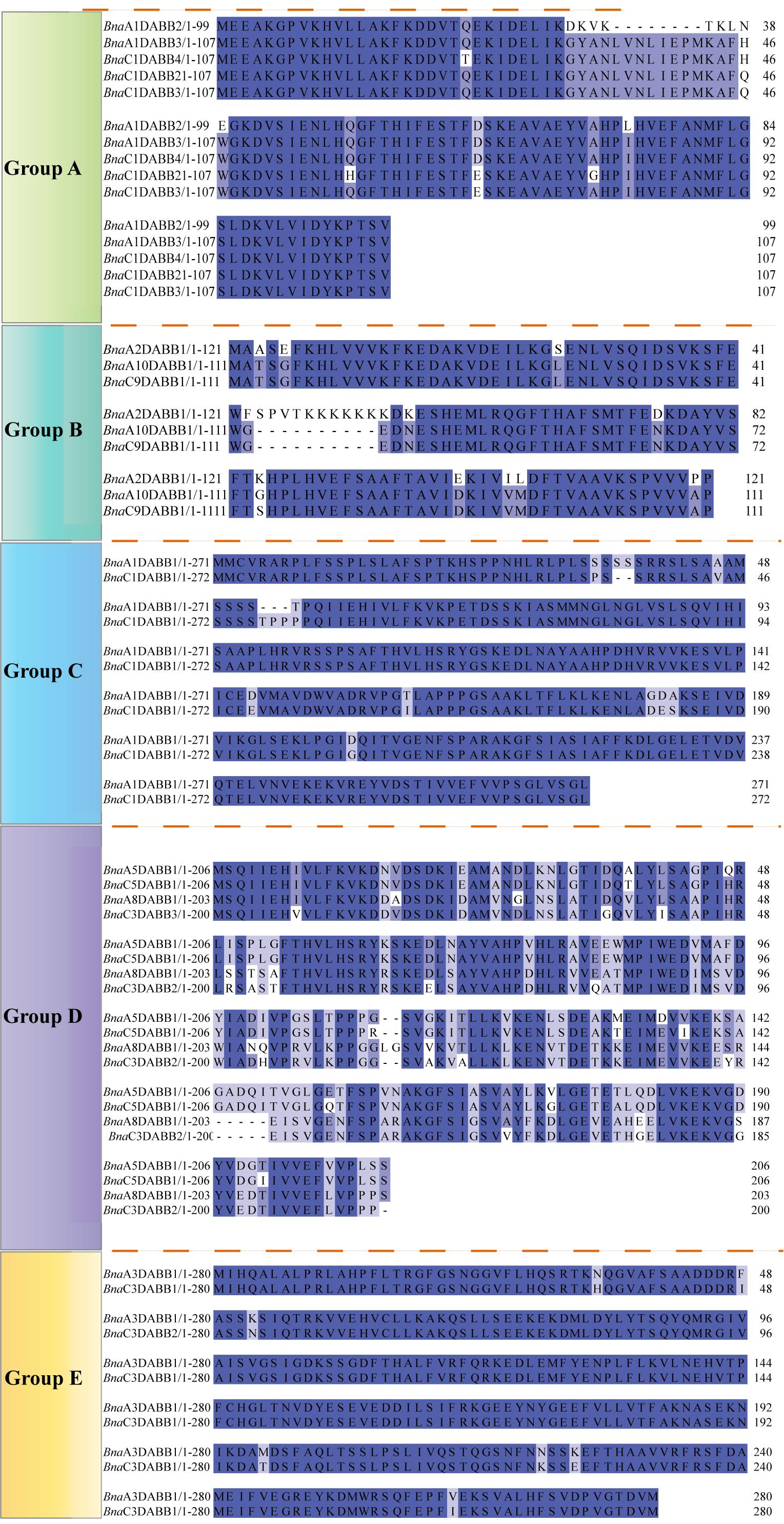

Supplement: Supplementary file 1 [file ijms-25-05721-s001.zip › Figure S2.jpg]

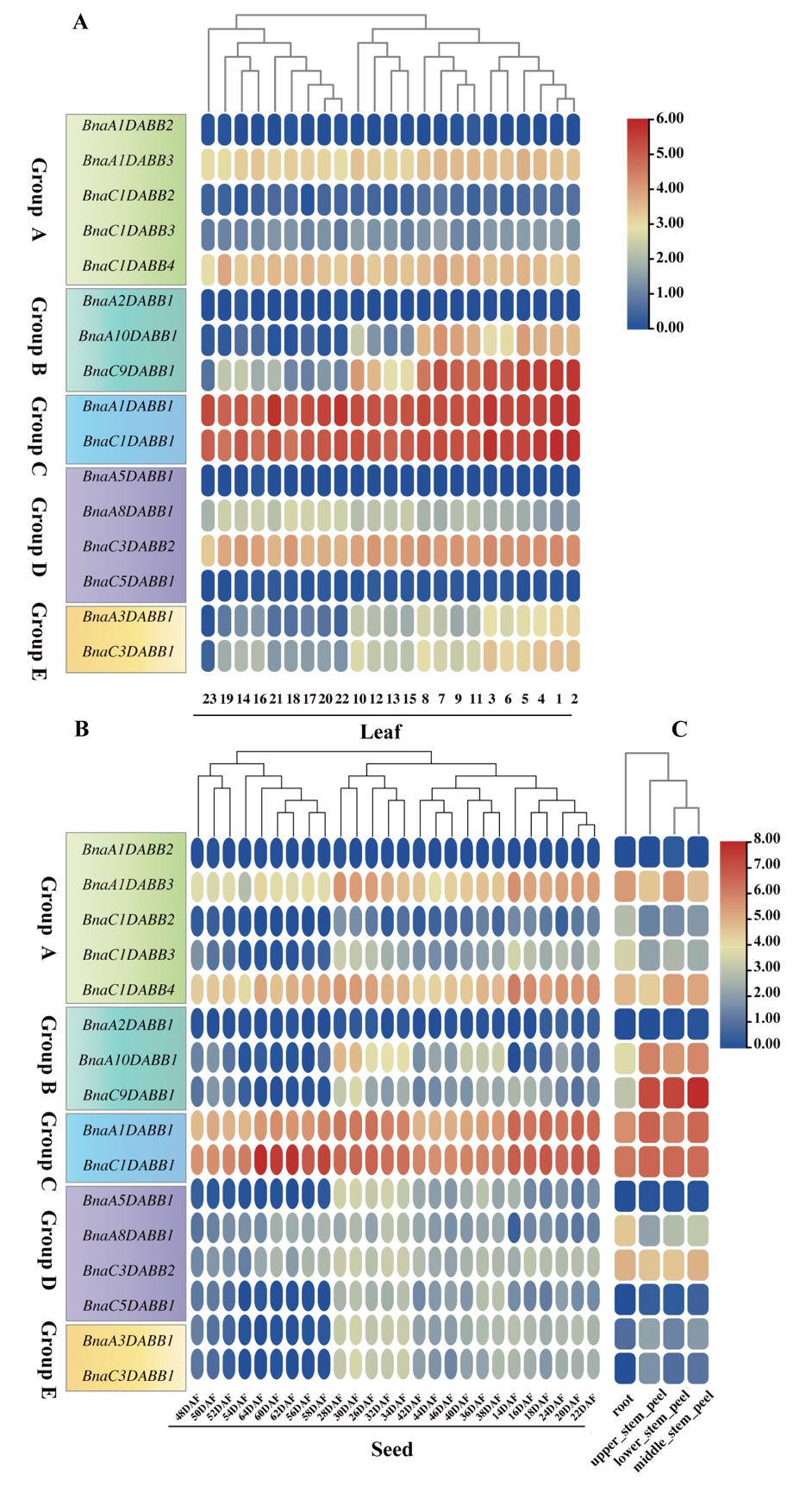

Supplement: Supplementary file 1 [file ijms-25-05721-s001.zip › Figure S3.jpg]

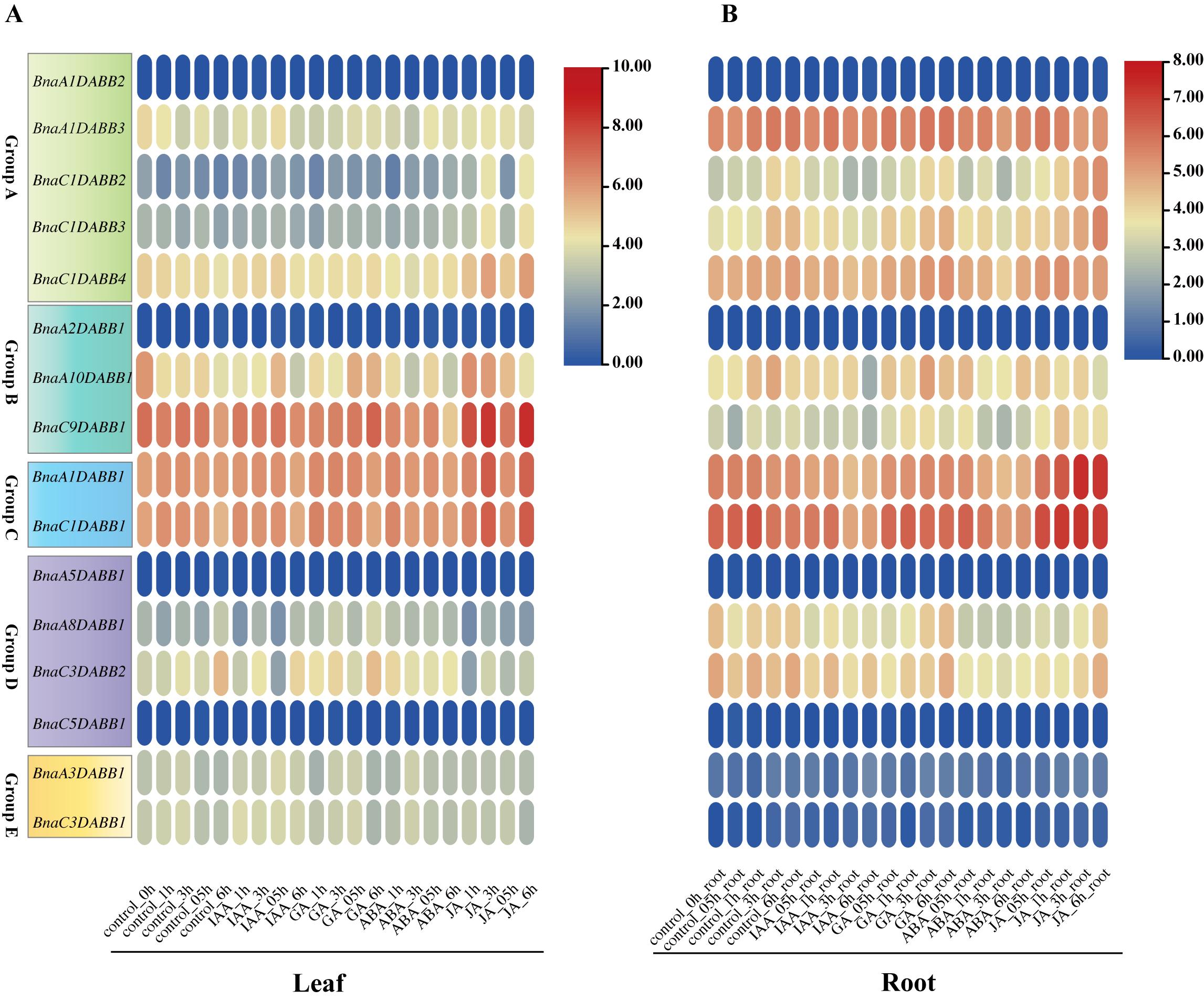

Supplement: Supplementary file 1 [file ijms-25-05721-s001.zip › Figure S4.jpg]

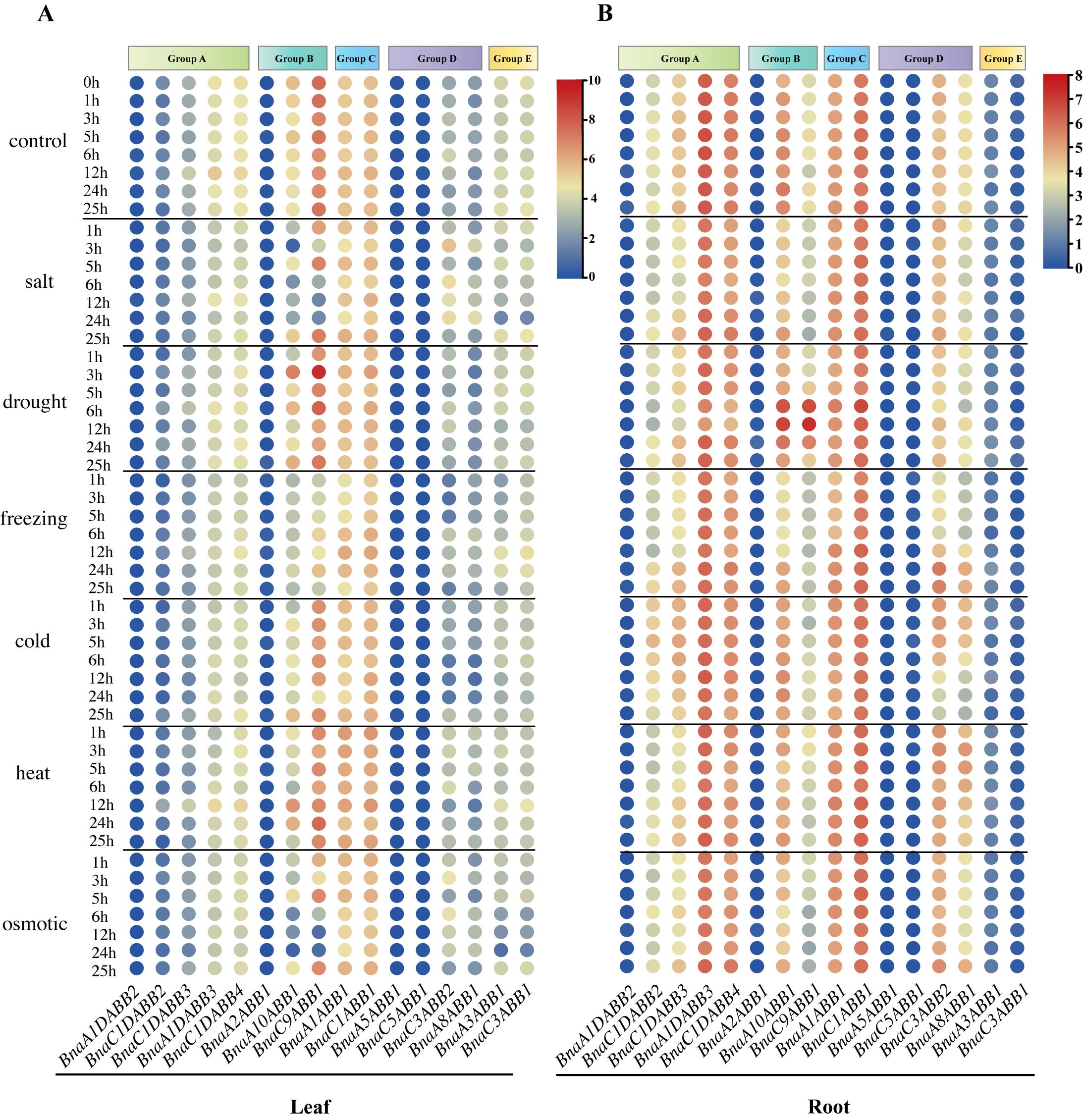

Supplement: Supplementary file 1 [file ijms-25-05721-s001.zip › Figure S5.jpg]
